# Chikungunya Virus in *Aedes albopictus*, Italy

**DOI:** 10.3201/eid1405.071144

**Published:** 2008-05

**Authors:** Paolo Bonilauri, Romeo Bellini, Mattia Calzolari, Raffaella Angelini, Luciano Venturi, Francesca Fallacara, Paolo Cordioli, Paola Angelini, Claudio Venturelli, Giuseppe Merialdi, Michele Dottori

**Affiliations:** * Istituto Zooprofilattico della Lombardia e dell’Emilia Romagna “B. Umbertini” Brescia, Italy; † Centro Agricoltura Ambiente "G.Nicoli," Crevalcore, Italy; ‡ Azienda Unità Sanitaria Locale, Ravenna, Italy; § Servizio di Sanità Pubblica Regione, Emilia Romagna, Italy; ¶ Azienda Unità Sanitaria Locale, Cesena, Italy

**Keywords:** Chikungunya, Italy, Aedes albopictus, letter

## Abstract

Chikungunya Virus in *Aedes albopictus*, Italy

**To the Editor:** Chikungunya virus (CHIKV) infection is a self-limiting illness characterized by fever, headache, weakness, rash, and arthralgia. Some patients show prolonged weakness or arthralgia lasting several months. In 2006, several Indian Ocean states and India experienced outbreaks of CHIKV infection, where the vector was postulated to be *Aedes albopictus* in at least some areas (*1**,**2*).

Starting from mid July 2007, in 2 villages in Ravenna Province in Italy, Castiglione di Ravenna (≈1,700 inhabitants) and Castiglione di Cervia (≈2,000 inhabitants), several residents sought treatment at local hospitals and health centers for high fever and arthralgia, joint and muscular pain, severe headaches, body aches, and in some cases, rash. Since the beginning of August 2007, an increasing number of febrile syndromes associated with arthralgia have been recorded among the residents of the area. By the end of August, the number of sick persons had increased to ≈150 (*3*). At the beginning of September, the disease was confirmed as chikungunya fever by the Superior Institute of Health (*4*).

On August 21 and 22, 2007, an entomologic investigation was carried out in the area. *Ae. albopictus* (215 females and 57 males), *Culex pipiens* (369 females and 15 males), and a few specimens of *Ae. caspius* (5 females) and *Anopheles* spp. (2 females) were collected by using 3 light traps without CO_2_ (Centers for Disease Control and Prevention [CDC], Atlanta, GA, USA) and 8 CO_2_-baited traps (similar to the CDC light trap) activated once overnight. Collections were obtained by using 2 small-handled aspirators per day of sampling. Collected mosquitoes were divided by species and pooled as described in the Table.

**Table:** Pooled mosquito samples analyzed for chikungunya virus, Italy

Pool	No. mosquitoes	Sex	Species	Site of collection	PCR results
1	125	F	*Aedes albopictus*	Castiglione Ravenna	Positive
2	90	F	*Ae. albopictus*	Castiglione Cervia	Positive
3	214	F	*Culex pipiens*	Castiglione Ravenna	Negative
4	155	F	*Cx. pipiens*	Castiglione Cervia	Negative
5	5	F	*A. caspius*	Castiglione Ravenna and Castiglione Cervia	Negative
6	2	F	*Anopheles* spp.	Castiglione Ravenna and Castiglione Cervia	Negative
7	57	M	*Ae. albopictus*	Castiglione Ravenna and Castiglione Cervia	Negative
8	15	M	*Cx. pipiens*	Castiglione Ravenna and Castiglione Cervia	Negative

Each pool was analyzed for CHIKV or nucleic acid (viral isolation and CHIKV-specific reverse transcription–PCR [RT-PCR]). Total RNA was extracted from supernatant of mosquitoes homogenized in minimal essential medium by using TRIzol LS (Invitrogen, Carlsbad, CA USA), and cDNA was synthesized by using SuperScript II (Invitrogen) and random primers. Two PCR protocols were used on the same samples: a nested RT-PCR (*5*) and a real-time PCR (*6*). Positive results were obtained from samples 1 and 2 (Table) with both PCR protocols. No positive control was available to the authors at the time of the first PCR-positive detection of virus from mosquitoes; therefore, laboratory contamination can be excluded. Moreover, from the same PCR-positive samples of *Ae. albopictus* (Table), viral isolation was achieved.

The 172-bp PCR fragment obtained after the second round of the nested RT-PCR (*5*) was located in the E2 gene between nt positions 9486 and 9660 according to nucleotide sequence of the S27 strain genome (GenBank accession no. AF369024). The sequence of this fragment was obtained by using a BigDye Terminator Cycle Sequencing kit (Applied Biosystems, Foster City, CA, USA) and compared with the available CHIKV sequences, including Indian isolates obtained during 2005–2006 (IND-06), the earlier Indian isolates (1963 and 1973), the isolate from Yawat in 2000, Reunion isolates during 2005–2006 (RU), the Senegal strain (1983), and the S27 and Ross isolates (1952). The sequence of the isolate from Italy is clustered into the Central/East African genotype and showed highest nucleotide identity (99.4%–100.0%) with isolates from IND-06 and 100% identity at the amino acid level with isolates from IND-06 and from RU. These results must be considered carefully because the short-sequenced fragment does not enable confirmation of the epidemiologic origin of the isolate from Italy. The sequence of the whole genome of CHIKV isolates from mosquitoes in Italy is an ongoing process; when completed, results will be available in GenBank.

The remainder of insect samples, which included a few male samples of *Ae. albopictus*, generated PCR-negative results with both protocols tested. Male mosquitoes were tested to detect evidence of transovarian transmission, but the small number of mosquitoes tested suggests possible vertical transmission of the virus in the Italian outbreak. Chikungunya fever in Italy has been reported recently by Beltrame et al. (*7*) but those cases involved 9 patients who were infected while traveling in regions where CHIKV was endemic.

In spite of the large diffusion of *Ae. albopictus* in Italy recorded since 1990 and broadly distributed all over the country (*8*), this outbreak of Chikungunya fever is evidence of an active endogenous circulation of the virus and could represent a possible introduction of this disease in Italy. No prediction can be made about spread and persistence of the virus in Italy because the vector is now present in all areas of the country and recent winters have been characterized by mild temperatures. Most likely, because transovarial infection has not been demonstrated for CHIKV, spread of infection will remain limited. However, as this report documents, Italy is at risk for infection with arboviruses, such as dengue virus and West Nile virus, which have serious effects on public health.
